# Supplementary material for: Endemism shapes viral ecology and evolution in globally distributed hydrothermal vent ecosystems
Source: Nat Commun. 2025 May 1;16:4076. doi: 10.1038/s41467-025-59154-x (PMC12043954; doi:10.1038/s41467-025-59154-x)
Supplement: Supplementary file 1 — Supplementary Information [file 41467_2025_59154_MOESM1_ESM.pdf]

Endemism shapes viral ecology and evolution in globally distributed hydrothermal vent ecosystems

Marguerite V. Langwig[1,2], Faith Koester[1], Cody Martin[1,3], Zhichao Zhou[1], Samantha B. Joye[4], Anna-Louise Reysenbach[5], Karthik Anantharaman[1,6,7*]

[1] Department of Bacteriology, University of Wisconsin-Madison, Madison, WI, USA
[2] Freshwater and Marine Sciences Program, University of Wisconsin-Madison, Madison, WI, USA
[3] Microbiology Doctoral Training Program, University of Wisconsin-Madison, Madison, WI, USA
[4] Department of Marine Sciences, University of Georgia, Athens, GA, USA
[5] Department of Biology, Portland State University, Portland, OR, USA
[6] Department of Integrative Biology, University of Wisconsin-Madison, Madison, WI, USA
[7] Department of Data Science and AI, Wadhwani School of Data Science and AI, Indian Institute of Technology Madras, Chennai, TN, India

Supplementary Notes

Supplementary Note 1. Viruses in the 65 geographically distinct clusters are predominantly predicted to be low-quality. Because these viruses have low estimates of genome completeness, we wanted to understand which regions of the viruses overlap and confirm that they are biologically meaningful. In a cluster of three low-quality, putatively lytic viruses from the Lau Basin and Brothers Volcano, the viral genomes overlap up to 21 kb at 93.2% identity, and this spans a minor tail protein and major capsid protein (Supplementary Data 6 and 7). In more distant sites, a viral pair reconstructed from the plumes of Axial Seamount and Guaymas Basin, respectively, shared 6.1 kb at 99.7% identity, and this region included six VOGs, one of which is a tail assembly chaperone. In another cluster, two short, low-quality viruses from the Mid-Atlantic Ridge and Brothers Volcano share 96.6% identity across a 2.9 kb region, which includes two baseplate proteins. Thus, even among low-quality viruses, we find support for shared viral genomic regions between geographically separated vents.

Supplementary Note 2. In plume samples, predictions within phylum Pseudomonadota are largely to Gammaproteobacteria, Alphaproteobacteria, and Bacteroidota. Predicted Gammaproteobacteria hosts span a range of genera, including *Colwellia*, *Acinetobacter*, *Thioglobus*, and *Alteromonas*, all of which are known to occur in hydrothermal plumes and seawater[1–4]. Predicted Alphaproteobacteria hosts include families Pelagibacteraceae (SAR11) and Sphingomonadaceae, supporting previous findings of the dominance of these organisms in seawater and hydrothermal plumes[5]. Similarly, most Bacteroidota host predictions are to the family Flavobacteriaceae, which has been identified in hydrothermal plumes with abundant extracellular peptidase genes, used to acquire carbon and nitrogen from the environment[6,7]. Compared to 1,131 predictions to bacterial hosts in the plume, there are 87 predictions to archaeal hosts and more than half of these are predictions to orders Nitrososphaerales, Pacearchaeales, and Poseidoniales. These orders have previously been identified as dominant archaea in hydrothermal plumes and deep seawater[8–10]. Pacearchaea in Guaymas Basin hydrothermal plumes have been characterized as having high connectivity with other microbial

groups based on metabolic networks, and are predicted to receive the greatest benefits from community interactions, including cellobiose, oxygen, carbon dioxide, and sulfide[10]. Microorganisms in the order Nitrososphaerales are known to widely encode *amoA* for ammonia oxidation (Thaumarchaea, formerly Marine Group I)[11] and Poseidoniales (formerly Marine Group II) that reside in the deep ocean are predicted to reduce nitrate, though are still largely understudied[8].

Viruses are predicted to infect a greater diversity and larger number of microbial phyla in hydrothermal deposits compared to plumes (6,591 host predictions in deposits, Supplementary Table 2). Most host predictions within phylum Pseudomonadota are to Campylobacterota (1,679), Gammaproteobacteria (685), and Alphaproteobacteria (675). More than half of the predictions to Campylobacterota are within the genera *Sulfurovum*, *Sulfurimonas*, and a genus with no cultured representatives, *UBA1140*. Cultured *Sulfurovum* and *Sulfurimonas* isolates from hydrothermal vents are known to be chemolithoautotrophic with the ability to oxidize sulfur and hydrogen and reduce sulfur, nitrate, and thiosulfate[12–14]. Among Gammaproteobacteria, some viruses are predicted to infect host genera that were also found in the plume, including *Colwellia*, *Acinetobacter*, and *Alterimonas*, however, the number of predictions to these microorganisms were much smaller. In deposits, predicted Gammaproteobacteria hosts largely include unknown genera, *Thiolapillus*, *Cocleimonas*, *Thiogranum*, and *Thiomicrorhabdus*. All of these bacterial genera have cultured representatives isolated from hydrothermal vents or deep sea sediments and are capable of chemolithoautotrophic sulfur oxidation[15–18]. Finally, Alphaproteobacteria host predictions are also largely to unknown or undescribed genera (*UBA5972* and *UBA3077*), as well as *Profundibacter* and *Thermopetrobacter*. *Profundibacter* was originally isolated from Loki's Castle vent field and is known to be piezophilic and anaerobic[19], while *Thermopetrobacter* was cultured from the Eastern Lau Spreading Center and is an aerobe capable of chemoautotrophic growth on hydrogen[20].

Of the 6,591 viral host predictions in deposit samples, 875 are attributed to Archaea, largely to phyla Thermoproteota (348), Methanobacteriota_B (195), Halobacteriota (111), and Thermoplasmatota (77). Thermoproteota are known to be common in deep-sea hydrothermal vent archaeal communities, though they are underrepresented in genomic databases[21]. In our dataset, nearly half of the Thermoproteota predictions are within families Acidilobaceae and Desulfurococcaceae. Acidilobaceae have recently been amended to include eight genera and range from acidophilic to neutrophilic thermo- or hyperthermophiles that use carbohydrates or protein-rich carbon for growth[21]. The Desulfurococcaceae family are also hyperthermophiles, capable of growing heterotrophically by sulfur respiration of organic compounds or chemolithoautotrophic sulfur reduction with hydrogen as the electron donor[22]. Nearly all virus-host predictions to phylum Methanobacteriota_B are within the *Thermococcus* genus (173/193). These archaea are ubiquitous in hydrothermal vents, where they are known to be sulfur-reducing hyperthermophiles and have the ability to use mixed heterotrophic and carboxydotrophic metabolism[23]. Among Halobacteriota, many host predictions are to the closely related *Archaeoglobus_B*, *Archaeoglobus_C*, and *Geoglobus* genera. Cultured isolates of *Archaeoglobus_B* and *Archaeoglobus_C* are strict anaerobic hyperthermophiles that use sulfur compounds as terminal electron acceptors, but are distinguished by an incomplete Wood-Ljungdhal pathway and inability to reduce sulfate, respectively[24]. The *Geoglobus* genus is represented by two cultured isolates, isolated from the Guaymas Basin and the Mid-Atlantic

Ridge, which are hyperthermophilic, anaerobic, and Fe(III)-reducing archaea[25]. Thermoplasmatota host predictions are mostly to undescribed genera, with the exception of *Aciduliprofundum*, whose sole cultured representative was isolated from hydrothermal vents and is an anaerobic heterotrophic sulfur- and iron-reducing thermoacidophile[26].

Supplementary Notes References

1. Sylvan, J. B., Pyenson, B. C., Rouxel, O., German, C. R. & Edwards, K. J. Time-series analysis of two hydrothermal plumes at 9°50'N East Pacific Rise reveals distinct, heterogeneous bacterial populations. *Geobiology* **10**, 178–192 (2012).
2. Sheik, C. S. *et al.* Spatially resolved sampling reveals dynamic microbial communities in rising hydrothermal plumes across a back-arc basin. *ISME J.* **9**, 1434–1445 (2015).
3. Dede, B. *et al.* Niche differentiation of sulfur-oxidizing bacteria (SUP05) in submarine hydrothermal plumes. *ISME J.* **16**, 1479–1490 (2022).
4. Yoon, J.-H., Kim, I.-G. & Oh, T.-K. Acinetobacter marinus sp. nov. and Acinetobacter seohaensis sp. nov., isolated from sea water of the Yellow Sea in Korea. *J. Microbiol. Biotechnol.* **17**, 1743–1750 (2007).
5. Li, J. *et al.* Distribution and Succession of Microbial Communities Along the Dispersal Pathway of Hydrothermal Plumes on the Southwest Indian Ridge. *Front. Mar. Sci.* **7**, 581381 (2020).
6. Li, M., Jain, S. & Dick, G. J. Genomic and Transcriptomic Resolution of Organic Matter Utilization Among Deep-Sea Bacteria in Guaymas Basin Hydrothermal Plumes. *Front. Microbiol.* **7**, 1125 (2016).
7. Nguyen, T. T. H., Myrold, D. D. & Mueller, R. S. Distributions of Extracellular Peptidases Across Prokaryotic Genomes Reflect Phylogeny and Habitat. *Front. Microbiol.* **10**, 413 (2019).
8. Rinke, C. *et al.* Correction: A phylogenomic and ecological analysis of the globally abundant Marine Group II archaea (Ca. Poseidoniales ord. nov.). *ISME J.* **14**, 878 (2020).
9. Dick, G. J. & Tebo, B. M. Microbial diversity and biogeochemistry of the Guaymas Basin deep-sea hydrothermal plume. *Environ. Microbiol.* **12**, 1334–1347 (2010).
10. Kuppa Baskaran, D. K., Umale, S., Zhou, Z., Raman, K. & Anantharaman, K. Metagenome-based metabolic modelling predicts unique microbial interactions in deep-sea

hydrothermal plume microbiomes. *ISME Commun* **3**, 42 (2023).

11. Dick, G. J. *et al.* The microbiology of deep-sea hydrothermal vent plumes: ecological and biogeographic linkages to seafloor and water column habitats. *Front. Microbiol.* **4**, 124 (2013).
12. Inagaki, F., Takai, K., Nealson, K. H. & Horikoshi, K. Sulfurovum lithotrophicum gen. nov., sp. nov., a novel sulfur-oxidizing chemolithoautotroph within the epsilon-Proteobacteria isolated from Okinawa Trough hydrothermal sediments. *Int. J. Syst. Evol. Microbiol.* **54**, 1477–1482 (2004).
13. Mino, S. *et al.* Sulfurovum aggregans sp. nov., a hydrogen-oxidizing, thiosulfate-reducing chemolithoautotroph within the Epsilonproteobacteria isolated from a deep-sea hydrothermal vent chimney, and an emended description of the genus Sulfurovum. *Int. J. Syst. Evol. Microbiol.* **64**, 3195–3201 (2014).
14. Han, Y. & Perner, M. The globally widespread genus Sulfurimonas: versatile energy metabolisms and adaptations to redox clines. *Front. Microbiol.* **6**, 989 (2015).
15. Nunoura, T. *et al.* Physiological and genomic features of a novel sulfur-oxidizing gammaproteobacterium belonging to a previously uncultivated symbiotic lineage isolated from a hydrothermal vent. *PLoS One* **9**, e104959 (2014).
16. Bai, S., Xu, H. & Peng, X. Microbial Communities of the Hydrothermal Scaly-Foot Snails From Kairei and Longqi Vent Fields. *Front. Mar. Sci.* **8**, 764000 (2021).
17. Mori, K., Suzuki, K.-I., Yamaguchi, K., Urabe, T. & Hanada, S. Thiogranum longum gen. nov., sp. nov., an obligately chemolithoautotrophic, sulfur-oxidizing bacterium of the family Ectothiorhodospiraceae isolated from a deep-sea hydrothermal field, and an emended description of the genus Thiohalomonas. *Int. J. Syst. Evol. Microbiol.* **65**, 235–241 (2015).
18. Tan, X.-Y. *et al.* Thiomicrorhabdus marina sp.nov., an obligate chemolithoautotroph isolated from tidal zone sediment, and genome insight into the genus Thiomicrorhabdus. *Front. Mar. Sci.* **10**, 1144912 (2023).

19. Le Moine Bauer, S. *et al.* Profundibacter amoris gen. nov., sp. nov., a new member of the Roseobacter clade isolated from Loki's Castle Vent Field on the Arctic Mid-Ocean Ridge. *Int. J. Syst. Evol. Microbiol.* **69**, 975–981 (2019).
20. Sislak, C. D. Novel Thermophilic Bacteria Isolated From Marine Hydrothermal Vents. (Portland State University, Ann Arbor, United States, 2013).
21. St John, E. & Reysenbach, A.-L. Genomic comparison of deep-sea hydrothermal genera related to Aeropyrum, Thermodiscus and Caldisphaera, and proposed emended description of the family Acidilobaceae. *Syst. Appl. Microbiol.* **47**, 126507 (2024).
22. Huber, H. *et al.* Ignicoccus gen. nov., a novel genus of hyperthermophilic, chemolithoautotrophic Archaea, represented by two new species, Ignicoccus islandicus sp nov and Ignicoccus pacificus sp nov. and Ignicoccus pacificus sp. nov. *Int. J. Syst. Evol. Microbiol.* **50 Pt 6**, 2093–2100 (2000).
23. Lee, H. S. *et al.* The complete genome sequence of Thermococcus onnurineus NA1 reveals a mixed heterotrophic and carboxydotrophic metabolism. *J. Bacteriol.* **190**, 7491–7499 (2008).
24. Slobodkina, G. *et al.* Physiological and Genomic Characterization of a Hyperthermophilic Archaeon sp. nov. Isolated From a Deep-Sea Hydrothermal Vent Warrants the Reclassification of the Genus. *Front. Microbiol.* **12**, 679245 (2021).
25. Slobodkina, G. B., Kolganova, T. V., Querellou, J., Bonch-Osmolovskaya, E. A. & Slobodkin, A. I. Geoglobus acetivorans sp. nov., an iron(III)-reducing archaeon from a deep-sea hydrothermal vent. *Int. J. Syst. Evol. Microbiol.* **59**, 2880–2883 (2009).
26. Reysenbach, A.-L. *et al.* A ubiquitous thermoacidophilic archaeon from deep-sea hydrothermal vents. *Nature* **442**, 444–447 (2006).

Supplementary Figures

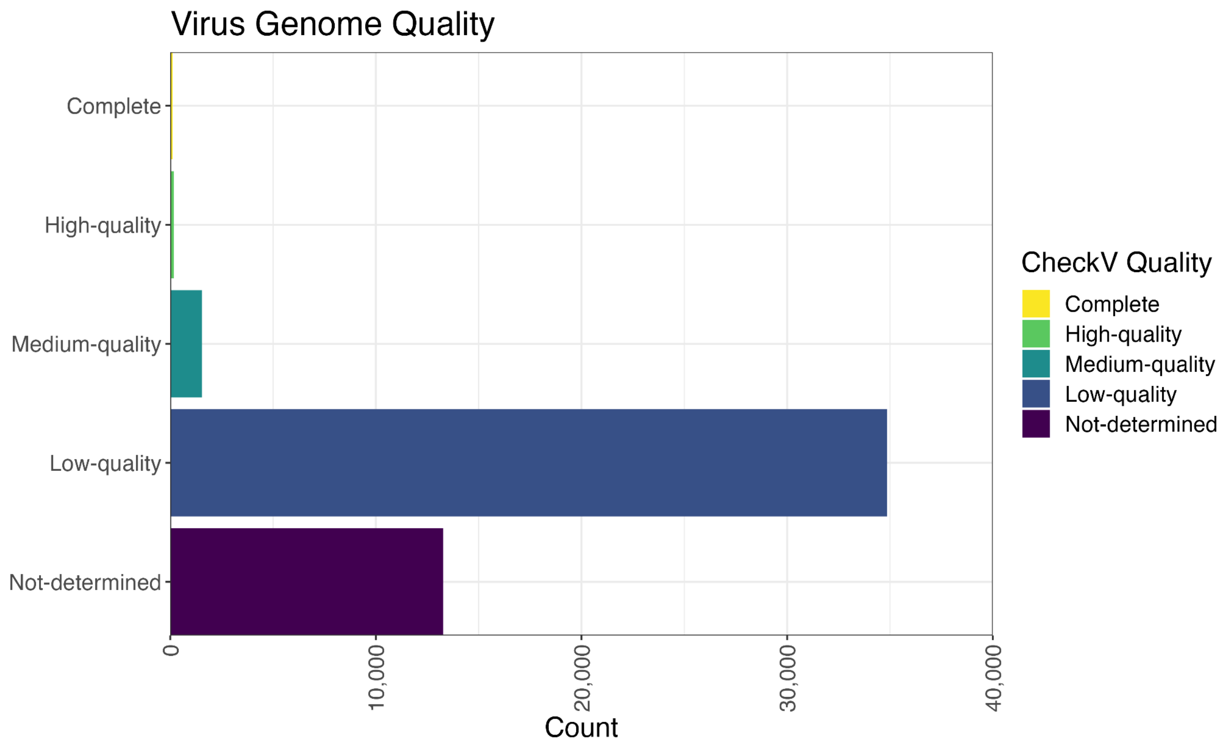

Supplementary Figure 1. Genome quality estimates of the 49,962 hydrothermal vent viruses according to CheckV. This figure was generated in R (VentVirus_Analysis2.R).

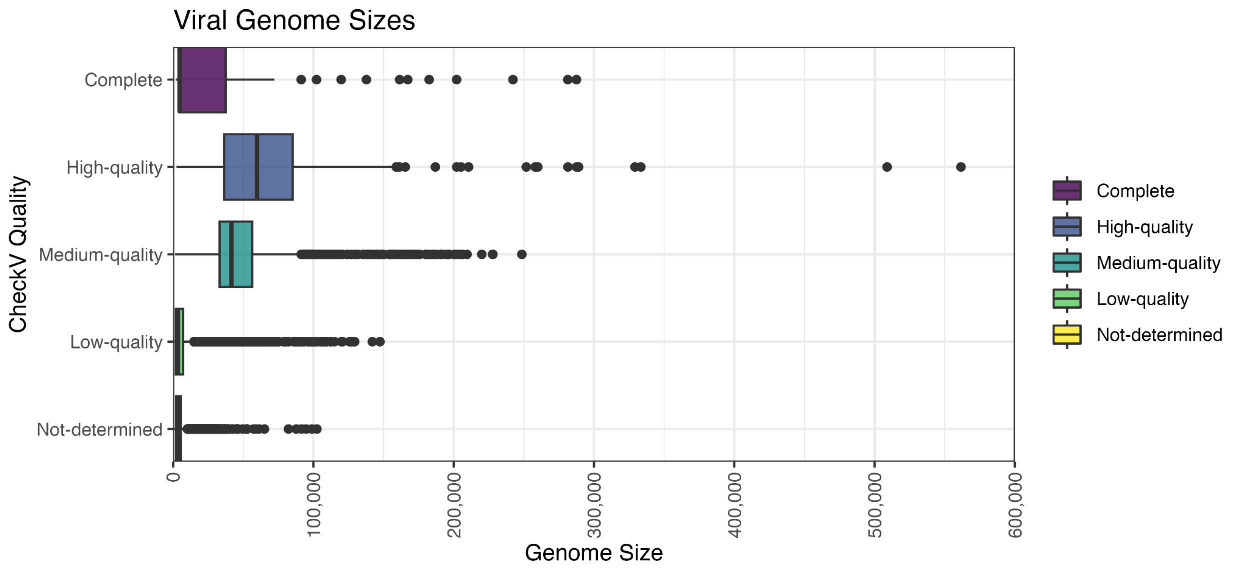

Supplementary Figure 2. Virus genome size distribution for each quality estimate category. Viral genome size was determined using seqkit. This figure was generated in R (VentVirus_Analysis2.R).

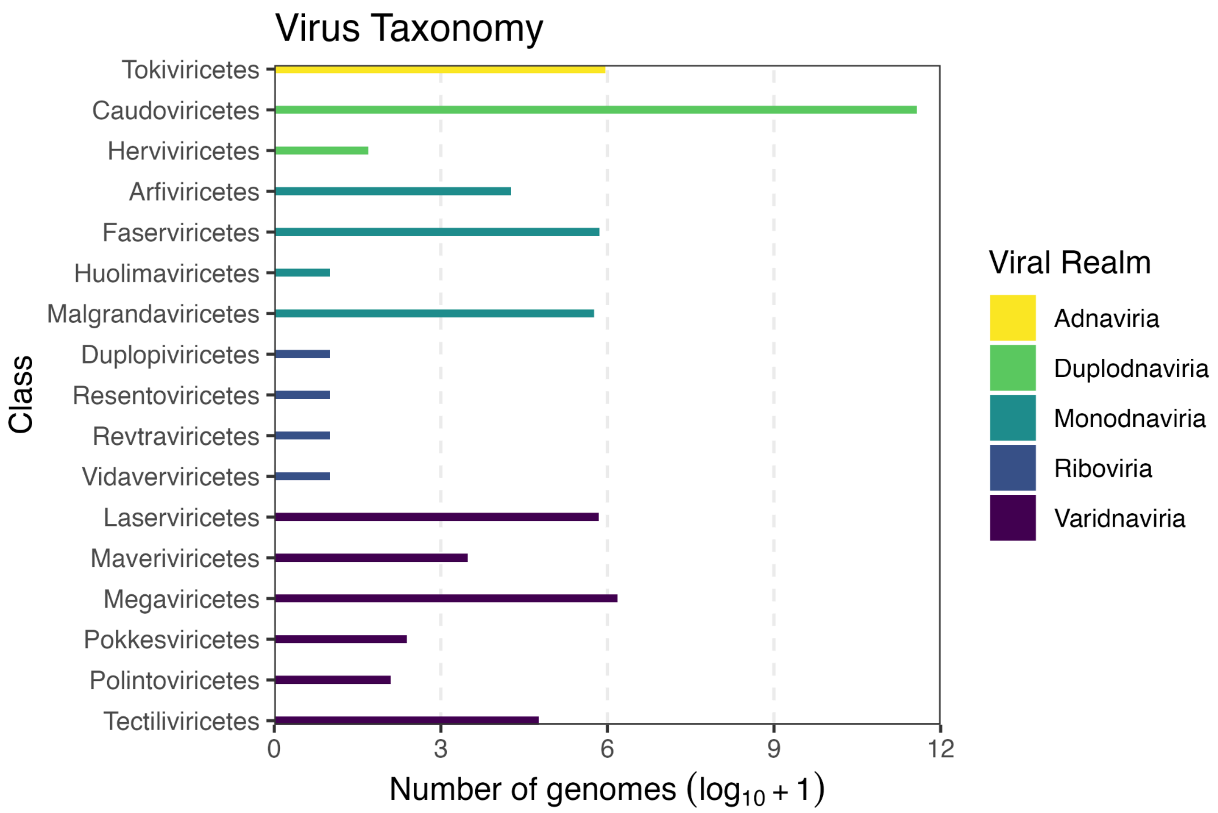

Supplementary Figure 3. Virus taxonomy of 40,236 vent viruses. The x axis shows the log+1-transformed number of viral genomes in each class. Bars are colored according to the realm of each class. Viruses that were assigned to an unknown realm and/or class are not included (392 viral genomes). This figure was generated in R (VentVirus_Analysis2.R).

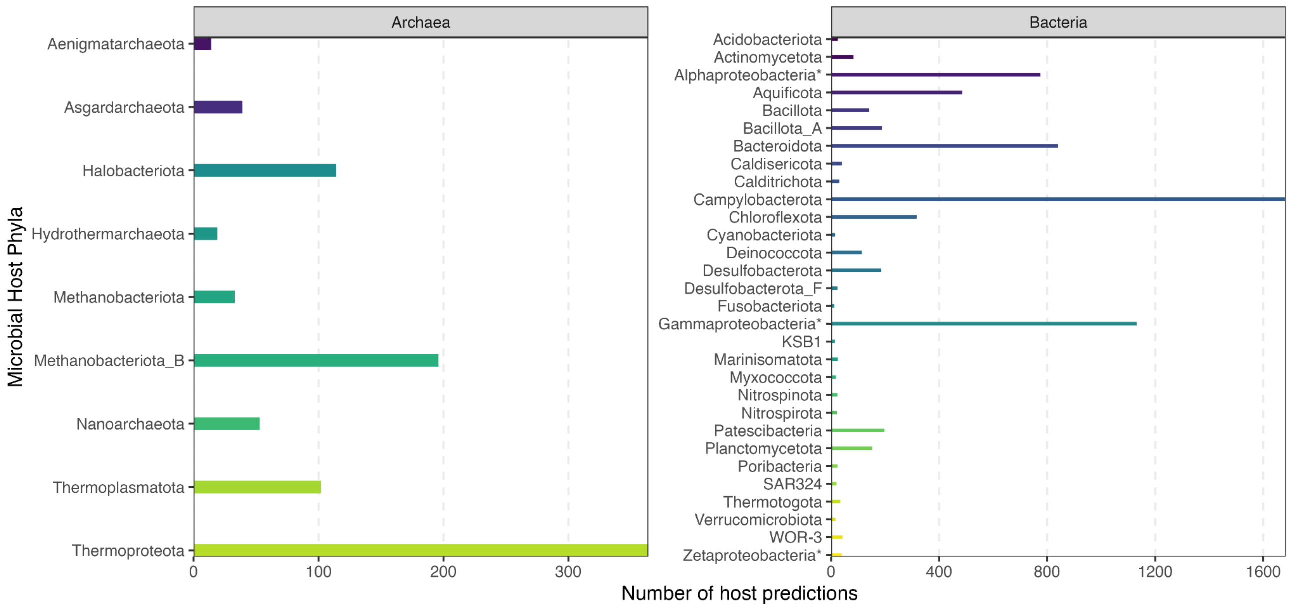

Supplementary Figure 4. Microbial host predictions for 7,001 hydrothermal vent viruses. Microbial phyla are shown on the y axis and the total number of hosts are shown on the x axis. Plots are faceted according to the microbial domains archaea and bacteria. Host predictions were made using iPHoP. This figure was generated in R (VentVirus_Analysis2.R).
