# Supplementary material for: Endemism shapes viral ecology and evolution in globally distributed hydrothermal vent ecosystems
Source: Nat Commun. 2025 May 1;16:4076. doi: 10.1038/s41467-025-59154-x (PMC12043954; doi:10.1038/s41467-025-59154-x)
Supplement: Supplementary file 3 — Reporting Summary [file 41467_2025_59154_MOESM3_ESM.pdf]

nature portfolio

Corresponding author(s): Karthik Anantharaman

Last updated by author(s): Mar 18, 2025

Reporting Summary

Nature Portfolio wishes to improve the reproducibility of the work that we publish. This form provides structure for consistency and transparency in reporting. For further information on Nature Portfolio policies, see our Editorial Policies and the Editorial Policy Checklist.

Statistics

For all statistical analyses, confirm that the following items are present in the figure legend, table legend, main text, or Methods section.

n/a	Confirmed	
☐	☒	The exact sample size (*n*) for each experimental group/condition, given as a discrete number and unit of measurement
☒	☐	A statement on whether measurements were taken from distinct samples or whether the same sample was measured repeatedly
☒	☐	The statistical test(s) used AND whether they are one- or two-sided<br>*Only common tests should be described solely by name; describe more complex techniques in the Methods section.*
☒	☐	A description of all covariates tested
☒	☐	A description of any assumptions or corrections, such as tests of normality and adjustment for multiple comparisons
☒	☐	A full description of the statistical parameters including central tendency (e.g. means) or other basic estimates (e.g. regression coefficient) AND variation (e.g. standard deviation) or associated estimates of uncertainty (e.g. confidence intervals)
☒	☐	For null hypothesis testing, the test statistic (e.g. *F*, *t*, *r*) with confidence intervals, effect sizes, degrees of freedom and *P* value noted<br>*Give P values as exact values whenever suitable.*
☒	☐	For Bayesian analysis, information on the choice of priors and Markov chain Monte Carlo settings
☒	☐	For hierarchical and complex designs, identification of the appropriate level for tests and full reporting of outcomes
☒	☐	Estimates of effect sizes (e.g. Cohen's *d*, Pearson's *r*), indicating how they were calculated

Our web collection on statistics for biologists contains articles on many of the points above.

Software and code

Policy information about availability of computer code

Data collection	Data collection involved oceanographic cruises to obtain deep sea samples that were then sequenced to obtain metagenomic assemblies. All of the reads used to generate assemblies are publicly available, and the SRR or ENA IDs of the reads are available in Supplementary Data 1.
Data analysis	The software used to analyze the data include the following: MEGAHIT v1.1.2, MetaBAT v0.32.4 , DAS Tool v1.0, CheckM v1.0.7, FastQC v0.11.8 , metaSPAdes v3.12.0 , and v3.11.1, MetaWRAP v1.2.2 , VIBRANT v1.2.1, vRhyme v1.1.0, BWA-MEM v0.7.17, samtools v1.7, SeqKit v2.6.1, R v4.4.0, geNomad v1.5.1, CheckV v0.8.1, skani v0.2.0, Vskani v0.0.1, BLASTN v2.14.1, bedtools v2.31.0, Mmseqs2 v15.6f452, PHROGS v4.0, CoverM v0.6.1, iPHoP v1.3.3. Code used to analyze the data is publicly available on GitHub at https://github.com/mlangwig/HydrothermalVent_Viruses

For manuscripts utilizing custom algorithms or software that are central to the research but not yet described in published literature, software must be made available to editors and reviewers. We strongly encourage code deposition in a community repository (e.g. GitHub). See the Nature Portfolio guidelines for submitting code & software for further information.

Data

Policy information about availability of data

All manuscripts must include a data availability statement. This statement should provide the following information, where applicable:

- Accession codes, unique identifiers, or web links for publicly available datasets
- A description of any restrictions on data availability
- For clinical datasets or third party data, please ensure that the statement adheres to our policy

The viral genomes recovered in this study are available at https://figshare.com/articles/dataset/Hydrothermal_Vent_Viruses/25968037. The predicted medium-, high-quality, and complete viruses with geNomad-assigned taxonomy are available through NCBI BioProject ID PRJNA1183900 [https://www.ncbi.nlm.nih.gov/bioproject/PRJNA1183900]. The microbial metagenome-assembled genomes are publicly available through NCBI BioProject IDs PRJNA488180 [https://www.ncbi.nlm.nih.gov/bioproject/PRJNA488180/] and PRJNA821212 [https://www.ncbi.nlm.nih.gov/bioproject/PRJNA821212/]. All the raw reads are publicly available with the SRA and ENA ID numbers listed in Supplementary Data 1.

Research involving human participants, their data, or biological material

Policy information about studies with human participants or human data. See also policy information about sex, gender (identity/presentation), and sexual orientation and race, ethnicity and racism.

Reporting on sex and gender	*Use the terms sex (biological attribute) and gender (shaped by social and cultural circumstances) carefully in order to avoid confusing both terms. Indicate if findings apply to only one sex or gender; describe whether sex and gender were considered in study design; whether sex and/or gender was determined based on self-reporting or assigned and methods used. Provide in the source data disaggregated sex and gender data, where this information has been collected, and if consent has been obtained for sharing of individual-level data; provide overall numbers in this Reporting Summary. Please state if this information has not been collected. Report sex- and gender-based analyses where performed, justify reasons for lack of sex- and gender-based analysis.*
Reporting on race, ethnicity, or other socially relevant groupings	*Please specify the socially constructed or socially relevant categorization variable(s) used in your manuscript and explain why they were used. Please note that such variables should not be used as proxies for other socially constructed/relevant variables (for example, race or ethnicity should not be used as a proxy for socioeconomic status). Provide clear definitions of the relevant terms used, how they were provided (by the participants/respondents, the researchers, or third parties), and the method(s) used to classify people into the different categories (e.g. self-report, census or administrative data, social media data, etc.) Please provide details about how you controlled for confounding variables in your analyses.*
Population characteristics	*Describe the covariate-relevant population characteristics of the human research participants (e.g. age, genotypic information, past and current diagnosis and treatment categories). If you filled out the behavioural & social sciences study design questions and have nothing to add here, write "See above."*
Recruitment	*Describe how participants were recruited. Outline any potential self-selection bias or other biases that may be present and how these are likely to impact results.*
Ethics oversight	*Identify the organization(s) that approved the study protocol.*

Note that full information on the approval of the study protocol must also be provided in the manuscript.

Field-specific reporting

Please select the one below that is the best fit for your research. If you are not sure, read the appropriate sections before making your selection.

☐ Life sciences ☐ Behavioural & social sciences ☒ Ecological, evolutionary & environmental sciences

For a reference copy of the document with all sections, see nature.com/documents/nr-reporting-summary-flat.pdf

Ecological, evolutionary & environmental sciences study design

All studies must disclose on these points even when the disclosure is negative.

Study description	We analyzed viral genomes identified in publicly available metagenomic data from deep-sea hydrothermal vents. Closely related viruses and their functions were identified at these sites using nucleotide and protein clustering methods. In addition, virus relative abundance and host prediction were used to link patterns in microbial abundance with the abundance of viruses that infect them.
Research sample	49,962 virus genomes, identified from publicly available metagenomic data reconstructed from deep-sea hydrothermal vents.
Sampling strategy	Samples were collected from 7 hydrothermal vent fields. Sample size was determined based on success of DNA extraction from field samples, and money available for next generation sequencing. Differing genomic assembly sizes were addressed when needed in the manuscript by, for example, normalizing data to the number of reads in a sample.

Data collection	Viruses were identified from publicly available metagenomic data using VIBRANT v1.2.1.
Timing and spatial scale	The dates and depths at which samples were collected are outlined in Supplementary Table 1. Data collection spans 14 years and 7 hydrothermal vents, dictated by ship availability and proposed dates/locations of sampling by the original sample collectors.
Data exclusions	No data were excluded from the analyses.
Reproducibility	All code used to analyze viral genomes is publicly available on GitHub, and all sequence data is publicly available with relevant IDs listed in the manuscript or supplementary information.
Randomization	This is not relevant to the study, because viruses were not allocated into groups for testing.
Blinding	Blinding was not possible due to the nature of the questions in this study - to understand how viruses compare between hydrothermal vents, it was necessary to know the location the viruses originated from during anaylsis.

Did the study involve field work? ☐ Yes ☒ No

Reporting for specific materials, systems and methods

We require information from authors about some types of materials, experimental systems and methods used in many studies. Here, indicate whether each material, system or method listed is relevant to your study. If you are not sure if a list item applies to your research, read the appropriate section before selecting a response.

Materials & experimental systems

n/a	Involved in the study	
☒	☐	Antibodies
☒	☐	Eukaryotic cell lines
☒	☐	Palaeontology and archaeology
☐	☒	Animals and other organisms
☒	☐	Clinical data
☒	☐	Dual use research of concern
☒	☐	Plants

Methods

n/a	Involved in the study	
☒	☐	ChIP-seq
☒	☐	Flow cytometry
☒	☐	MRI-based neuroimaging

Animals and other research organisms

Policy information about studies involving animals; ARRIVE guidelines recommended for reporting animal research, and Sex and Gender in Research

Laboratory animals	The study did not involve laboratory animals.
Wild animals	The study did not involve wild animals.
Reporting on sex	This information has not been collected.
Field-collected samples	Guaymas Basin plume samples were collected by “tow-yo” casts using a CTD rosette in 10 L Niskin bottles. This water was then filtered onto 142 mm 0.2 µm polycarbonate filters by N2 gas pressure filtration and preserved in RNAlater. Mid-Cayman plume samples were collected using a Suspended Particle Rosette Sampler (SUPR) by filtering 10-60 L plume water onto 142 mm 0.2 µm SUPOR membranes. These samples were then preserved in RNAlater in situ. In Lau Basin, SUPR-collected samples were filtered onto 0.2 and 0.8 µm pore size SUPOR polyethersulfone membranes in situ and preserved in RNAlater-flooded vials. In Axial Seamount, plume samples were collected by a Seabird SBE911 CTD and 10L Niskin bottles. After deposit samples were collected, they were subsampled on ship with the outer few millimeters (up to approximately 5 mm) kept separate from the bulk sample. These exterior samples were homogenized and stored at −80°C for subsequent DNA extraction.
Ethics oversight	No ethical approval or guidance was required. No human or animal samples were used in our study. All appropriate permits for sampling were obtained as part of the research cruises.

Note that full information on the approval of the study protocol must also be provided in the manuscript.

Plants

Seed stocks	No plant material was used.
Novel plant genotypes	No plant material was used.
Authentication	No plant material was used.
