# Supplementary material for: Endemism shapes viral ecology and evolution in globally distributed hydrothermal vent ecosystems
Source: Nat Commun. 2025 May 1;16:4076. doi: 10.1038/s41467-025-59154-x (PMC12043954; doi:10.1038/s41467-025-59154-x)
Supplement: Supplementary file 4 — Description of Additional Supplementary Files [file 41467_2025_59154_MOESM4_ESM.pdf]

Description of Additional Supplementary Files

File Name: Supplementary Data 1
Description: Metadata associated with all hydrothermal vent samples.

File Name: Supplementary Data 2
Description: Characteristics of the 49,962 viruses (e.g., number of hallmarks, lifestyle, CheckV quality). Columns r through f represent the geNomad taxonomy predictions of viruses, encompassing the realm, kingdom, phylum, class, order, and family predictions.

File Name: Supplementary Data 3
Description: CoverM relative abundance, filtered for viruses with read covered fraction ≥0.70. The abun norm is the read count normalized by the covered fraction and total number of reads in that sample, summed by site and viral class. The log n plus1 is this value log transformed plus 1. This table is the input used to create Figure 1B.

File Name: Supplementary Data 4
Description: Characteristics of all hydrothermal vent assemblies, determined using seqkit.

File Name: Supplementary Data 5
Description: The 866 clusters (column 'id') that contain viruses from distinct vent sites or sample types, identified using nucleotide clustering.

File Name: Supplementary Data 6
Description: Regions of overlap (identified with blastn) between low-quality viruses in the nucleotide clusters.

File Name: Supplementary Data 7
Description: Protein annotations of the regions of overlap in viruses in the nucleotide clusters.

File Name: Supplementary Data 8
Description: Coverm read mapping of reads that mapped to viruses from a different site or between sample types,filtered for ≥70% covered fraction and ≥3kb viruses.

File Name: Supplementary Data 9
Description: Nucleotide clustering results of the viruses recovered in this study compared to the GOV 2.0 dataset.

File Name: Supplementary Data 10
Description: Mmseqs protein clusters that are shared between geographically separated vents and between plume and deposit samples. Annotations are shown when identified, and represent the best hit to the protein from the VOG, KEGG, and pfam databases, searched by VIBRANT.

File Name: Supplementary Data 11
Description: PHROG anotations (v4.0) of viral proteins filtered by ≥75% identity, ≥80% coverage, and best hits obtained using lowest e-value and highest bit score.

File Name: Supplementary Data 12
Description: Abundance of viruses with a predicted microbial host. Normalized abundance was calculated as the number of reads mapped divided by the total number of reads for that sample.

File Name: Supplementary Data 13

Description: Predicted microbial hosts of viruses, determined using iPHoP.

File Name: Supplementary Data 14
Description: CoverM relative abundance of all microbial MAGs in all samples. The 'Taxonomy' column contains theGTDBtk (v2.3.2) taxonomy predictions of microbial MAGs.

File Name: Supplementary Data 15
Description: Proportionality between microbial and viral abundance (read counts) determined using the propr package in R (v5.1.4) with the metric rho and the centered log-ratio transformation.

File Name: Supplementary Data 16
Description: Viral annotations supported as AMGs according to DRAMv.
